# Effect of Different Pressures on Microstructure and Mechanical Performance of F-III Fibers in Supercritical Carbon Dioxide Fluid

**DOI:** 10.3390/ma12050690

**Published:** 2019-02-26

**Authors:** Xiaoma Ding, Haijuan Kong, Mengmeng Qiao, Zhifeng Hu, Muhuo Yu

**Affiliations:** 1 State Key Laboratory for Modification of Chemical Fibers and Polymer Materials, College of Materials Science and Engineering, Donghua University, Shanghai 201620, China; 1159124@mail.dhu.edu.cn (X.D.); 2160320@mail.dhu.edu.cn (M.Q.); 2160226@mail.dhu.edu.cn (Z.H.); 2 School of Materials Engineer, Shanghai University of Engineer Science, Shanghai 201620, China; Konghaijuan@sues.edu.cn

**Keywords:** F-III fibers, mechanical performance, supercritical carbon dioxide fluid, wide-angle X-ray scattering, small-angle X-ray scattering

## Abstract

F-III fibers were treated at different pressures in supercritical carbon dioxide fluid and all samples including untreated and treated F-III fibers were characterized by a mechanical performance tester, wide-angle X-ray scattering and small-angle X-ray scattering. By studying the relationship between mechanical performance and microstructural changes of the samples, it was found that microstructural change was the main cause of variation in mechanical performance. Results revealed that the maximum tensile strength and modulus of F-III fibers were acquired at 14 MPa within the pressure range of 8 MPa to 16 MPa when the temperature, tension and time were 250 °C, 6 g·d^−1^ and 40 min, respectively. Correspondingly, the microstructures of the samples, including the phase fraction, crystal size, orientation factor, fibril radius, fibril length and misorientation angle, have been investigated. It was fortunate that the supercritical carbon dioxide fluid could be used as a medium during the hot-stretch process to improve the mechanical performance of F-III fibers, although the treatment temperature was lower than the glass transition temperature of the F-III fibers.

## 1. Introduction

The discovery of high-performance product named aramid fibers (AFs) has brought infinite vitality to military, aerospace, electronic appliances, cables, sports equipment, fiber reinforced matrix composites and other excavated and unexcavated application fields [1,2,3,4]. A lot of literature has revealed that the mechanical performance of AFs can be greatly enhanced by post-processing such as hot-stretch treatment [5,6,7,8,9]. However, for the sake of achieving desired performance, it is significant to understand how different post-processing conditions will affect the structure of AFs and which structure corresponds to good performance [10]. As a new kind of AFs, the F-III fibers with high crystallinity [11,12,13,14] and excellent performance can be further strengthened by hot-stretch treatment. Owing to these special properties, F-III fibers are specially used in the field of bulletproofing.

Hot-stretch treatment of AFs has been studied by many researchers in the last few decades. In this process, the improvement of the mechanical performance of AFs mainly lies in the amelioration of the microstructure. For instance, Ran and Lee [7,8] pointed out that tensile strength and modulus of AFs increased with the increase in crystallinity under a certain tension during the hot-stretch process. In addition, it is well known that treatment time is very short in air and nitrogen atmospheres, however, the treatment temperature is particularly high, which will largely destroy the internal structure of AFs so that full performance is not well demonstrated. Furthermore, it is worth mentioning that the mechanical performance of AFs has no obvious changes when the treatment temperature is lower than the glass transition temperature (Tg) [7], which is attributed to the insignificant movement of the molecular chains.

Supercritical carbon dioxide (Sc-CO_2_) fluid is famous for its excellent swelling and plasticizing characteristics [15,16,17,18]. It can not only be used as a green and environment-friendly medium for extraction or carrying small molecules [19,20,21], however, it can also be employed as a kind of heating atmosphere for a series of hot-stretch treatments [22,23,24]. Kong et al. have successfully utilized Sc-CO_2_ fluid to carry the hexamethylene diisocyanate into the amorphous regions of AFs to improve tensile strength and modulus [19]. Hobbs et al. found that the presence of Sc-CO_2_ fluid could enhance tensile strength and elastic modulus of polyethylene terephthalate fibers [22,23]. More importantly, most molecular segments of fibers are allowed to move below Tg due to the existence of Sc-CO_2_ fluid, which is not available in other media [25]. Therefore, it provides a solid basis for us to treat F-III fibers by way of hot-stretch in Sc-CO_2_ fluid at temperatures lower than the Tg of F-III fibers.

A lot of literature has reported the relationship between mechanical performance and the microstructure of AFs during the hot-stretch process in atmospheres such as air and nitrogen. However, research on the application of Sc-CO_2_ fluid in this area is still relatively rare. In this work, Sc-CO_2_ fluid was tentatively employed as a hot-stretch medium to treat AFs. Additionally, the influence of different pressures of Sc-CO_2_ fluid on the mechanical performance of AFs was studied, and the corresponding microstructure was also systematically characterized.

## 2. Materials and Methods

### 2.1. Materials

The untreated F-III fiber made up of 150 monofilaments with the denier of 44 tex and the Tg of 275 °C was provided by Inner Mongolia Aerospace New Materials Technology Co., Ltd., Inner Mongolia, China. Carbon dioxide (CO_2_) with a purity of 99.99% was purchased from Sinopharm Chemical Reagent Co., Ltd., Shanghai, China.

### 2.2. Preparation of Hot-Stretch Samples in the Sc-CO_2_ Reactor

**Figure 1:**
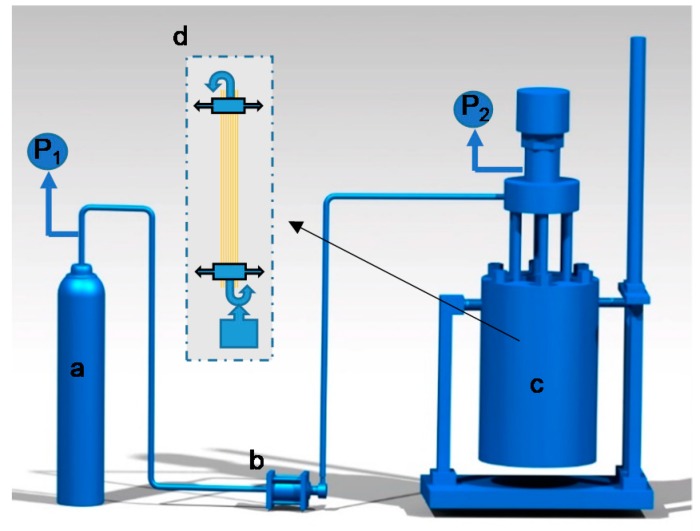
Schematic diagram of the hot-stretch treatment device. a: carbon dioxide cylinder; b: booster pump; c: high-pressure reactor; d: tension applicator.

Figure 1 shows a schematic diagram of the hot-stretch treatment device. The Sc-CO_2_ reactor with a volume of 10 L was manufactured by Tianjin Yantu Experimental Instrument Development Co., Ltd., Tianjin, China. The booster pump with a pressure ratio of 60 was allowed to provide the desired pressures for such a large reactor, and the pressure reducing valve at the outlet of the CO_2_ cylinder was used mainly to enable the output CO_2_ gas to enter the reactor at a constant rate. Both ends of each untreated F-III fiber, which had a length dimension of nearly 200 mm, were locked with the self-designed mechanical interlocking device. The weights were vertically hung at one end of the mechanical interlocking device with a hook, and the other end was suspended inside the top cover of the reactor, as shown in part d of Figure 1. During the experiment, the reactor was heated to a temperature of 250 °C, set to a pressure of approximately 2 MPa with CO_2_ gas, then the exhaust valve was opened to remove the air in the reactor, and finally, different pressures were applied through multiple experiments. The reaction lasted 40 min with the tension of 6 g·d^−1^ when the temperature was maintained at 250 °C and the pressure was controlled at 8 MPa, 10 MPa, 12 MPa, 14 MPa and 16 MPa, respectively, as required.

### 2.3. Characterization of Samples

Wide-angle X-ray scattering (WAXS) measurement was performed on a beam line (BL14B) at Shanghai Synchrotron Radiation Facility. The wavelength dimension of the X-ray was 0.124 nm and the distance between the detector (Mar 345) and the sample was 120.5 mm. The crystal, mesomorphic and amorphous fractions of each sample were acquired from the 2D WAXS pattern analyzed by Xpolar software which was purchased from Precision Machinery Co., Ltd., NY, USA. The average crystallite size was calculated according to the Scherrer equation reported by Gupta [26], as shown in Equation (1):

(1) $$L_{\mathrm{hkl}}=\frac{k\lambda }{\beta \cos\theta }$$

where L_hkl_ is the crystallite size of the crystal plane perpendicular to (hkl) reflections, the value of the constant k is 1, λ is 0.124 nm, β is the integral width of the reflection peak and θ is the half value of the diffraction angle. Similarly, the orientation factor of specific crystal planes was referenced in Herman’s equation reported by Yu et al. [27], as presented in Equation (2):

(2) $$f_{c}=\frac{3\cos^{2}\omega -1}{2}$$

where f_c_ is the orientation factor of the crystal plane, ω is the angle between the c-axis crystal unit and the fiber axis, (cos^2^ω) is the orientation parameter for the reflection (110), and (cos^2^ω_110_) can be determined by Equation (3):

(3) $$\cos^{2}\omega_{110}=\frac{\int_{0}^{\frac{\pi }{2}}I\left(\beta_{110}\cos^{2}\beta_{110}{\sin\beta }_{110}{d\beta }_{110}\right)}{\int_{0}^{\frac{\pi }{2}}I\left(\beta_{110}{\sin\beta }_{110}{d\beta }_{110}\right)}$$

where β_110_ is the azimuthal angle of (110) reflection and I (β_110_) is the intensity of the azimuthal angle of (110) reflection.

Small-angle X-ray scattering (SAXS) measurement was performed on a beam line (BL14B) with the same wavelength dimension as WAXS. The distance between the detector (Mar CCD 165) and the sample was 1950 mm, which was different from the WAXS measurement. The resulting analysis of the SAXS pattern was also performed by Xpolar software and the radiuses of scattering objects in F-III fibers were calculated according to the paper written by Guinier and Fournet [28], as revealed in Equation (4):

(4) $$I\left(q\right)=I\left(0\right)\exp\left(-\frac{q^{2}R^{2}}{5}\right)$$

where I(q) is the intensity and R is the radius of the scattering object. The parameter q is obtained using q = 4πsinθ/λ. The fibril length and misorientation angle were analyzed according to the method proposed by Ruland [29], as depicted in Equation (5):

(5) $$s^{2}B_{\mathrm{obs}}^{2}=\frac{1}{l_{f}^{2}}+s^{2}B_{\varphi }^{2}$$

where l_f_ is the length of the fibrils in F-III fiber, B_φ_ is the angle between the scattering object and the fiber axis, s is the scattering object, and s is acquired using s = 2sin θ/λ, B_obs_ is the full width at the half maximum of the azimuthal profile.

Mechanical performance including tensile strength, modulus, and elongation at break was tested using a single-filament strength tester named XQ-1A (Shanghai New Fiber Instrument Co., Ltd., Shanghai, China) which had a gauge length of 20 mm and a speed of 10 mm·min^−1^ at the College of Textile, Donghua University, Shanghai, China. The average values of these parameters were chosen from at least 30 effective data. The units of tensile strength and modulus were converted using Equation (6):

(6) $$\mathrm{GPa}=0.1\times \rho \times \left(\mathrm{cN}\cdot {\mathrm{dtex}}^{-1}\right)$$

where Gpa and cN·dtex^−1^ are two kinds of representations of tensile strength and modulus units. The value of ρ was 1.44 g·cm^−3^.

## 3. Results and Discussion

### 3.1. WAXS Analysis

**Figure 2:**
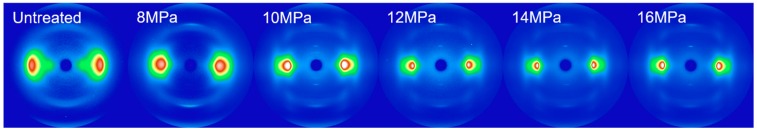
Wide angle X-ray scattering (WAXS) patterns of the untreated and treated F-III fibers in supercritical carbon dioxide (Sc-CO_2_) fluid.

Figure 2 shows the 2D WAXS patterns from all samples. The pattern of the untreated F-III fibers showed gourd-shaped diffraction spots in the equator, illustrating a relatively ordered intermolecular packing along the transverse fiber axis. Additionally, the clear diffraction halos appeared in the meridian, expounding a weak orientation along the fiber axis. Therefore, a 3D crystalline structure was composed of the diffraction spots, diffraction halos and some weak off-equatorial diffraction arcs. The equatorial diffraction spots became sharper and the meridional diffraction halos as well as the diffraction arcs in the off-equatorial direction turned clearer with the increase in pressure, indicating that the internal orientation of F-III fibers had improved in all directions. When the pressure was between 12 MPa and 16 MPa, the WAXS patterns showed no obvious differences, indicating a good treatment pressure range at the temperature of 250 °C, tension of 6 g·d^−1^ and a time of 40 min in Sc-CO_2_ fluid.

**Figure 3:**
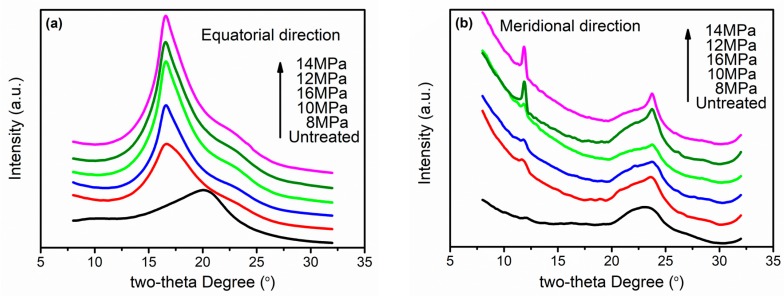
The 1D WAXS patterns of the untreated and treated F-III fibers in Sc-CO_2_ fluid: (**a**) Equatorial direction, (**b**) meridional direction.

The 1D WAXS patterns of all samples in the direction of equator and meridian are displayed in Figure 3a,b, respectively. Both peaks at 2θ = 20.3° in the equator and 2θ = 23.4° in the meridian of the untreated F-III fibers were wide, indicating a low crystallinity of the untreated F-III fibers. The position of the peak moved from 2θ = 20.3° to 2θ = 16.6° after hot-stretch treatment, implying an increase in interplanar spacing, as shown in Figure 3a. In addition, the peak at 2θ = 16.6° became sharper as the pressure increased, indicating the improvement of crystallinity in the equatorial direction. The curves of 1D WAXS patterns in the equator within the pressure range of 12 MPa to 16 MPa were similar, which corresponded to the 2D WAXS patterns above. To our surprise, the 1D WAXS curves of F-III fibers used in this experiment only exhibited a peak corresponding to the (110) crystal plane in the equatorial direction. This was mainly due to the special synthesis process of the untreated F-III fibers provided by the company. Additionally, two peaks at 2θ = 12.4° and 2θ = 24.3° corresponding to (002) and (004) crystal planes in the meridional direction, respectively, were acquired after hot-stretch treatment, and the sharpest curves of the peaks were acquired at both 12 MPa and 14 MPa.

**Figure 4:**
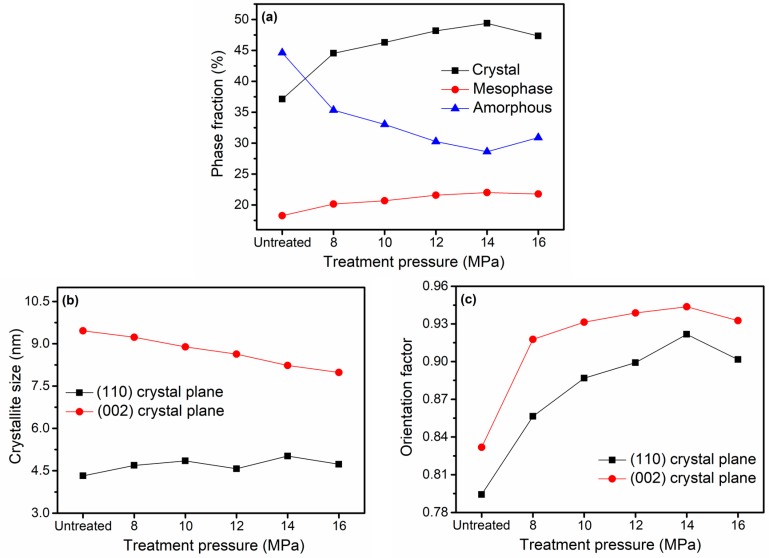
(**a**) The crystal, mesomorphic and amorphous fractions of the untreated and treated F-III fibers in Sc-CO_2_ fluid, (**b**) the crystalline sizes at (110) crystal plane in the equatorial direction and (002) crystal plane in the meridional direction, (**c**) the orientation factors of crystal in (110) and (002) crystal planes.

**Figure 5:**
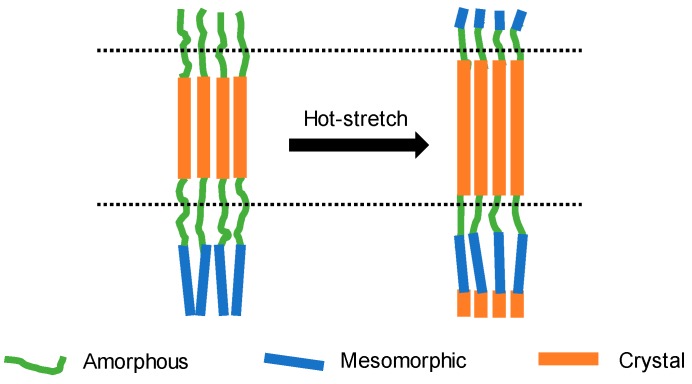
Schematic diagram of the possible phase transition of F-III fibers before and after hot-stretch treatment in Sc-CO_2_ fluid.

The qualitative calculations of phase fractions and crystallite sizes of (110) and (002) crystal planes as well as orientation factors of (110) and (002) crystal planes are shown in Figure 4. Compared with the untreated group, the treated F-III fibers presented higher crystallinities, and the crystallinity increased from 37.12% to 49.38% at 14 MPa, which was the best treatment pressure to acquire high crystallinity F-III fibers. This phenomenon was observed mainly because the Sc-CO_2_ fluid could dissolve in fiber interiors, increase the free volume of fibers [30], reduce the forces among chain segments and increase the flexibility of chain segments. Therefore, the Sc-CO_2_ fluid played a plasticizing role [31,32] and showed a good solvation effect [33,34,35]. Hence, the crystallinity of F-III fibers could be improved at temperatures below Tg owing to the presence of Sc-CO_2_ fluid [36]. However, the subsequent increase in pressure played a counteraction. The reason behind this can be summarized as follows: Static pressure was generated when the pressure of the system was too high, as a result, the free volume of fibers shrunk, and the movement of chain segments was limited, which increased the difficulty of movement among chain segments. Therefore, the plasticizing effect of Sc-CO_2_ fluid played a leading role within a certain pressure range, however, the static pressure could not be ignored beyond a certain pressure [37]. Crystal, intermediate, and amorphous phases were mainly observed in F-III fibers, and there were clear interfaces among them [38,39]. It can be seen that phase transitions in F-III fibers mainly converted from the amorphous phase to the crystal or intermediate phases within the pressure range, as the data show in Figure 4a. This process can also be intuitively understood from Figure 5.

The crystalline size of (110) crystal plane in the equatorial direction showed an upward trend within the pressure range, while the crystalline size of (002) crystal plane in the meridional direction decreased continuously. This was because the particles grew naturally without tension in the equator, while the particles were restricted under certain external forces along the meridian. On the whole, the crystal particles with large sizes tended to be acquired along the fiber axis. The variation tendency of particle sizes could also reflect the change trend of crystallinity to a certain extent. The orientation factors in Figure 4c reflected the degree of orientation of crystal in (110) and (002) crystal planes. The orientation factors along and perpendicular to the direction of the fiber axis increased as the pressure raised to 14 MPa and then started to decrease. The reason for this was that the plasticization effect of Sc-CO_2_ fluid was gradually enhanced as the pressure increased, and the molecular chains of fibers moved and rearranged along the direction of the external force, so the degree of orientation of crystal was gradually increased. However, the Sc-CO_2_ fluid would inhibit the movement of molecular chains when the pressure was too high.

### 3.2. SAXS Analysis

**Figure 6:**
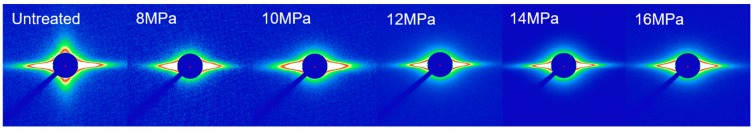
Small angle X-ray scattering (SAXS) patterns of the untreated and treated F-III fibers in Sc-CO_2_ fluid.

Figure 6 showed the microstructure of all samples analyzed by SAXS. Lotz and Cohen [40,41,42] elucidated that the equatorial and meridional reflections in SAXS patterns presented a bidirectional morphology, which was caused by the epitaxial growth of small crystals owing to the irregular arrangement. Unlike the untreated group, the SAXS patterns of treated F-III fibers only exhibited a streak in the equator. The streaks were sharper at high pressures, indicating a better orientation of scattering objects in F-III fibers. Regarding AFs, according to a lot of previous literature, the elongated streaks in the equator basically depended on two possibilities: Microfiber and void structures [43,44]. Grubb et al. suggested that the scattering objects in Kevlar-49 were principally related to microfiber structure [43], and according to the latest study of Luo et al. [39], they pointed out that the scattering objects in F-III fibers were also related to microfiber structure. In this paper, we were prone to be consistent with their conclusions combined with previous work in the summary and analysis of our related data.

**Figure 7:**
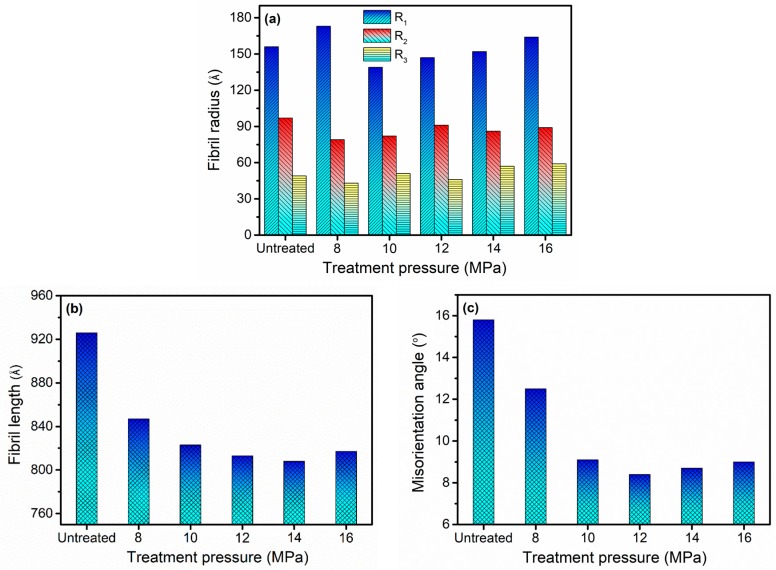
(**a**) The fibril radius, (**b**) fibril length and (**c**) misorientation angle of the untreated and treated F-III fibers in Sc-CO_2_ fluid. R_1_, R_2_ and R_3_ represent the fibril radius of different grades.

The fibril radius, fibril length and misorientation angle calculated from SAXS patterns are displayed in Figure 7. The radii of microfibers were approximately distributed between 43 Å and 173 Å as shown in Figure 7a. According to previous literature [45], this set of values could only roughly estimate the size distribution of microfibers in F-III fibers. The fibril length decreased with the increase in pressure ranging from 8 MPa to 14 MPa and then tended to increase at 16 MPa, as depicted in Figure 7b. The decrease in fibril length might be due to two possibilities: The movement of molecular chains in F-III fibers became more intense with the increase in pressure, resulting in the rupture of some long microfibers; the external compressive force on F-III fibers gradually increased as the pressure increased, causing some long microfibers to break. Later, the reason for the rising trend of fibril length was found to be due to excessive pressures which restricted the movement of molecular chains, which was mentioned previously in the WAXS analysis section. Compared with the untreated group, the treated F-III fibers possessed a smaller misorientation angle, and the minimum value was acquired at 12 MPa, implying the best orientation of fibrils in F-III fibers, as depicted in Figure 7c. Strictly speaking, the smallest misorientation angle could only be obtained at an appropriate pressure.

### 3.3. Mechanical Performance

**Figure 8:**
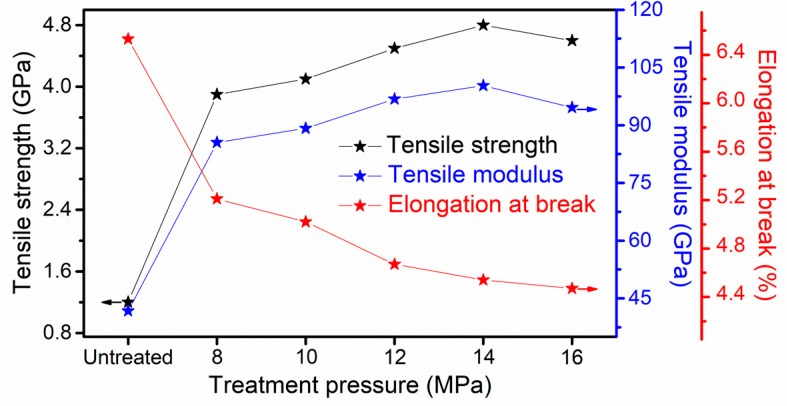
Tensile strength, tensile modulus and elongation at break of the untreated and treated F-III fibers in Sc-CO_2_ fluid.

The tensile strength, tensile modulus and elongation at break of all samples are presented in Figure 8. The tensile strength and modulus of the untreated F-III fibers were 1.2 GPa and 41.7 GPa, respectively, and both showed a rising trend with the increase in pressure of Sc-CO_2_ fluid. The maximum tensile strength and modulus were obtained at 14 MPa, which were 4.8 GPa and 100.3 GPa, respectively. Unfortunately, the tensile strength and modulus began to fall when the pressure rose to 16 MPa, and the change tendency of mechanical performance corresponded to the variation trend of crystallinity and degree of orientation. The elongation at break, as an important index to evaluate the toughness of fibers, is shown in Figure 8. The elongation at break decreased with the increase in pressure, indicating that the wildness of F-III fibers decreased with the increase in tensile strength and modulus.

## 4. Conclusions

In this paper, F-III fibers were treated with pressure ranging from 8 MPa to 16 MPa in Sc-CO_2_ fluid when the temperature, tension and time were 250 °C, 6 g·d^−1^ and 40 min, respectively. Throughout the full text, results showed that tensile strength increased from 1.2 GPa to 4.8 GPa and tensile modulus increased from 41.7 GPa to 100.3 GPa when the pressure was 14 MPa. The WAXS results showed that crystallinity increased from 37.12% to 49.38%, the orientation factor of crystal of (110) crystal plane increased from 79.43% to 92.17% and the orientation factor of crystal of (002) crystal plane increased from 83.18% to 94.37% at 14 MPa. Overall, Sc-CO_2_ fluid can be used as a medium to improve the mechanical performance of F-III fibers, although the temperature needs to be lower than the Tg of F-III fibers.
